# Risk and protective factors for opioid overdose during the COVID-19 pandemic

**DOI:** 10.1192/j.eurpsy.2021.2179

**Published:** 2021-08-13

**Authors:** J. Wong, J. Westenberg, N. Mathew, P. Azar, R.M. Krausz

**Affiliations:** 1 Psychiatry, Addictions and Concurrent Disorders Research Group, Institute of Mental Health, Vancouver, Canada; 2 Psychiatry, BC Mental Health and Substance Use Services, Burnaby, Canada; 3 Psychiatry, Vancouver General Hospital, Vancouver, Canada

**Keywords:** opioid overdose, COVID-19, safe supply, opioid overdose crisis

## Abstract

**Introduction:**

People who use drugs (PWUD) are now at the intersection of two public health emergencies – the Covid-19 pandemic and the overdose crisis. They may be at heightened risk of overdose due to increased isolation, worsened mental health, and changes to the illicit drug supply. The province of British Columbia (BC) in Canada is anticipated to experience a record-breaking year of overdose deaths as over 1,500 people (32.9 deaths per 100,000) have died from overdose in 2020. In response, BC released new clinical guidelines in March to allow the prescribing of pharmaceutical alternatives aiming to reduce PWUD’s risk of overdose and contracting Covid-19.

**Objectives:**

We examined the risk and protective factors for overdose during these dual crises. We explored how the Covid-19 pandemic has impacted the mental health and substance use of PWUD and their access to treatment and harm reduction services.

**Methods:**

We are conducting a survey among patients with opioid use disorder at a major hospital in Vancouver, BC. It includes the following domains: sociodemographic characteristics; mental and physical health; substance use patterns; opioid overdose history; access to treatment, harm reduction services; impacts of Covid-19.

**Results:**

We anticipate collecting data from 200 participants. Descriptive statistics and regression analysis will be conducted to describe the sample and determine the risk, protective factors for overdose.

**Conclusions:**

We will gain a better understanding of overdose risk in PWUD who are now navigating the complex challenges created by the dual crises. This will in turn inform the establishment of evidence-based strategies to reduce their overdose risk.

**Disclosure:**

No significant relationships.

